# eggNOG v7: phylogeny-based orthology predictions and functional annotations

**DOI:** 10.1093/nar/gkaf1249

**Published:** 2025-12-08

**Authors:** Ana Hernández-Plaza, Ziqi Deng, Fabian Robledo-Yagüe, Damian Szklarczyk, Christian von Mering, Peer Bork, Jaime Huerta-Cepas

**Affiliations:** Centro de Biotecnología y Genómica de Plantas, Universidad Politécnica de Madrid (UPM)—Instituto Nacional de Investigación y Tecnología Agraria y Alimentaria (INIA-CSIC), Campus de Montegancedo-UPM, 28223 Madrid, Spain; Centro de Biotecnología y Genómica de Plantas, Universidad Politécnica de Madrid (UPM)—Instituto Nacional de Investigación y Tecnología Agraria y Alimentaria (INIA-CSIC), Campus de Montegancedo-UPM, 28223 Madrid, Spain; Institute for Integrative Systems Biology, Spanish National Research Council (CSIC), 46980, Paterna, Spain; Swiss Institute of Bioinformatics and University of Zurich, Winterthurerstrasse 190, 8057 Zurich, Switzerland; Swiss Institute of Bioinformatics and University of Zurich, Winterthurerstrasse 190, 8057 Zurich, Switzerland; European Molecular Biology Laboratory, Meyerhofstrasse 1, 69117 Heidelberg, Germany; Department of Bioinformatics, Biocenter, University of Würzburg, G97074 WürzburgGermany; Centro de Biotecnología y Genómica de Plantas, Universidad Politécnica de Madrid (UPM)—Instituto Nacional de Investigación y Tecnología Agraria y Alimentaria (INIA-CSIC), Campus de Montegancedo-UPM, 28223 Madrid, Spain

## Abstract

The eggNOG (evolutionary genealogy of genes: Non-supervised Orthologous Groups) database is a phylogenomic resource for orthology inference, evolutionary analysis, and functional annotation across eukaryotes, bacteria, and archaea. Previous versions relied on best reciprocal hit triangulation and clustering approaches, which, although effective, faced challenges with the computational demands of large datasets, inconsistent hierarchical orthologous group (OG) reconstruction, and inaccurate classification of multidomain proteins. Here, we present eggNOG v7, the first release implementing a fully phylogenetic, domain-centric workflow. In this pipeline, sequences are first pre-clustered by Pfam domains or *de novo* clustering, followed by large-scale multiple sequence alignment and phylogenetic tree inference. Speciation and duplication events are then detected using a noise-tolerant algorithm to generate hierarchically consistent, evolutionarily dated OGs. Applied to 59.3 million proteins from 12 535 species, eggNOG v7 produced 3.18 million OGs, reducing singletons, fragmentation, and oversized groups compared to prior versions. Benchmarking against manually curated KEGG functional OGs demonstrated higher functional consistency. Additionally, eggNOG v7 provides updated protein functional annotations and a fully redesigned web interface with protein-centric searches, interactive phylogenies, and functional profiling tools. eggNOG v7 is available at https://eggnogdb.org.

## Graphical Abstract

**Graphical Abstract:**
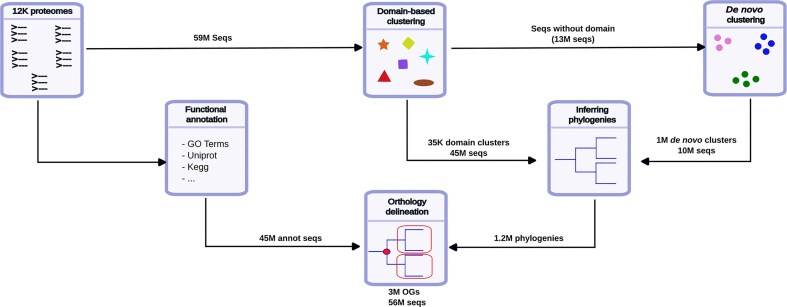

## Introduction

The eggNOG (evolutionary genealogy of genes Non-supervised Orthologous Groups) database is a phylogenomic resource that provides orthology relationships, phylogenetic analysis, and functional annotations for over 59 million proteins spanning a broad range of eukaryotic, bacterial, and archaeal species. eggNOG’s orthology predictions are commonly employed for the functional annotation of new proteomes, the profiling of metagenomes (i.e. via eggNOG-mapper [1]), and the characterization of the evolutionary history of specific gene families.

Previous versions of eggNOG ([2, 3]) inferred orthologous groups (OGs) using all-against-all best reciprocal hits (BRH) analysis, which was originally proposed in [4] and subsequently adopted and modified by other orthology resources such as the COG database [5], OrthoDB [6], OMA [7], MBGD [8], or Inparanoid [9]. In addition to BRH graph analysis, eggNOG predictions were refined through phylogenetic analysis, allowing the generation of pairwise orthology relationships between individual proteins and enabling the identification of in-paralogs and duplication events. This phylogeny-based approach is also commonly used in other resources such as Ensembl Compara [10], PhylomeDB [11], and Panther Database [12], as well as in bioinformatics software like Possvm [13] and OrthoFinder [14]. However, phylogeny-based predictions are typically limited by the number of species included due to computational and analytical reasons. Large phylogenetic trees in previous eggNOG versions often contain numerous artifacts that can affect the accurate detection of speciation and duplication events.

Thus, as datasets have become larger and more complex for orthology prediction methods [15], building the eggNOG database has become increasingly challenging from both conceptual and technical perspectives. First, the computational burden of calculating *de novo* BRH for tens of thousands of species has prevented eggNOG from adopting more agile updating cycles. Although fast BLAST-like software such as MMSeqs [16] and Diamond [17] reduced the problem, BRH calculations and network analysis remained highly demanding. Second, clustering-based OG delineation is not intended to accurately reconstruct the nested structure of OG groups at different taxonomic levels. Therefore, to provide orthology and functional information across most taxonomic groups, previous eggNOG pipelines reconstructed OGs independently at each taxonomic level of interest, and then their hierarchical structure was reconciled along the tree of life. However, this approach often resulted in inconsistencies that prevented clear reconstruction of the evolutionary history of OGs in terms of duplication events. Finally, clustering-based approaches tend to either leave distant sequences as separate groups (oversplitting) or produce massively large OGs, depending on parameters used. For example, eggNOG v6 produced 5164, 699 singletons (8.7% of all proteins covered), as well as 66 groups containing >100 000 proteins. The latter is actually a common outcome when dealing with multidomain proteins, which may contain highly promiscuous domains with complex evolutionary histories [18]. In fact, the large number of in-paralogs observed in these giant clusters suggests that a more accurate classification of such proteins would be possible by allowing for the distinct evolutionary histories of their protein domains to be reconstructed independently. This domain-aware orthology approach has been a matter of recent studies [18–20], revealing orthologous relationships not found by full-length sequence approaches [19].

Here, we present eggNOG v7, the first version of the database to adopt a fully phylogenetic, domain-centric approach to delineating OGs, which mitigates the aforementioned problems.

## Materials and methods

### Protein family reconstruction pipeline

Pfam searches were performed using v35 and the “*cut_ga*” parameter, which applies a gathering threshold to minimize false positives; while *de novo* clusters, were inferred using MMseqs2 [16] clustering mode with a minimum coverage and identity of 30%. To build the multiple sequence alignments, we used MAFFT [21] with default parameters for protein families with fewer than 1000 sequences and FAMSA [22] for those exceeding that number. For MAFFT, we used the “auto” parameter to automatically select the most suitable alignment algorithm. After alignment, an in-house trimming script (https://gist.github.com/jhcepas/59e858d5c49dc98d7926d99c00d3bfe6) was applied to remove columns with a gap content >90%. Finally, FastTree2 was used to reconstruct all phylogenies using default options.

### Noise tolerant orthology delineation algorithm

The delineation of orthologous and paralogous relationships involved analyzing phylogenetic tree topology using the Species Overlap (SO) score, an established algorithm [23], coupled with updated criteria for outlier detection. First, each phylogenetic tree was prepared by rooting it with the MinVar algorithm [24] and removing leaves with excessively long branches (defined as branches ≥ 50 times the mean branch length). Taxonomic information was subsequently assigned to all branches to allow for last common ancestor (LCA) calculation on each clade. To identify gene duplication events, we calculated the SO score for each internal node, classifying a node as a duplication if the score was ≥10%. To ensure the robustness of this calculation against technical artifacts or misplaced sequences (e.g. long-branch attraction or horizontal gene transfer), a two-step outlier exclusion method was applied prior to the final score calculation. Specific to eggNOG v7, this method involved first setting the lineage covering ≥90% of sequences within a node as the reference LCA lineage. Second, any sequence not belonging to this reference LCA was evaluated for taxonomic consistency: if any of the taxonomic groups represented by a candidate outlier sequence (i.e. any taxID in its NCBI lineage track) was better represented outside the current node (≥95% of sequences from that lineage were external), the sequence was classified as an outlier and temporarily excluded from the SO score calculation. Finally, for the purpose of OG delineation, only duplication nodes containing ≥70% of all sequences from the reference LCA were retained. Method implementation and benchmarks are available at https://github.com/AnaHrnndz/pbdood.

### Duplication rate calculations

The duplication rate per OG was calculated by averaging the number of duplication events detected in each OG phylogeny using ETE toolkit [25]. For this, only the phylogenies of basal OGs were considered, with species-specific duplication events being ignored. Thus, we scanned the phylogeny of each OG and identified nodes representing duplication events (i.e. with overlapping species between its two children branches). For the eggNOG v7 calculations, OG phylogenies were extracted as subtrees from its corresponding protein family tree. In eggNOG v6, we scanned the phylogenetic trees of all OGs at the bacterial, archaeal, and eukaryotic levels.

### Calculation of KEGG scores

Scores were determined by counting true positives (genes from a KO that were correctly assigned to the same OG), false positives (genes from a different KO that were incorrectly assigned to the same OG), and false negatives (genes from a KO that were incorrectly assigned to a different OG). For each KO, we selected the OG with the highest *f*-score as its equivalent.

### External database mappings

Protein domain annotation was performed using eggNOG-mapper and PFAM v35. Cross-references were integrated from UniProt (November 2024), PDB (May 2025), KEGG (July 2024), COG (2024 update), and BiGG (2019 update).

## Database improvements

### The new OG delineation workflow

The new eggNOG pipeline uses a combination of a domain-based pre-clustering approach and phylogenetically guided detection of speciation and duplication events to infer OGs at all taxonomic levels in a non-supervised manner. The method consists of the following steps:

The pipeline starts with mapping all target sequences in the database against the Pfam database, creating groups of protein sequences that consistently align with at least one known domain. These groups of sequences that share a Pfam domain are referred to as “protein families” from this point onwards. Multidomain proteins will therefore appear in as many families as the number of Pfam domains they contain, and all sequences within a protein family will contain at least one shared domain. This strategy prevents nonalignable proteins from being grouped together in the same cluster, as we previously observed occurring with protein families containing promiscuous domains (e.g. PTPLA or AlkA_N). Furthermore, it provides a domain-centric view of the orthology relationships within each set, enabling multidomain proteins to be recruited into different families. When extremely large clusters are found, which would likely prevent the application of phylogenetic reconstruction methods, our pipeline automatically splits the original clusters into subclusters using the *de novo* MMseqs clustering method. For the current version of eggNOG, subclustering was only necessary for the following protein domain families: Response_Reg, ABC_Tran, HATPase_C, Helicase_C, PKinase, and AMP-Binding. These families contained >180 000 proteins each and were split into 3185, 1663, 4772, 1292, 4744, and 1136 subclusters, respectively. Finally, all protein sequences without a detectable Pfam domain are clustered *de novo* based on MMseqs, producing a large pool of putative protein families. In total, the domain-based pre-clustering step in eggNOG v7 produced 35 072 Pfam-based clusters, which grouped 77% of all sequences, as well as 1 173 256 *de novo* clusters, which grouped 17.9% of proteins.

Next, to identify OGs within each protein family cluster, a multiple sequence alignment and a phylogenetic tree are inferred for each cluster. In this new version we aligned sequences using MAFFT [21] (<1000 sequences) or FAMSA [22] (larger protein sets), removed uninformative alignment columns using an *ad hoc* script (see Materials and methods), inferred phylogenetic trees using FastTree2 [26] and rooted using Minimum variance method [24] from the FastRoot package. OG-detection was performed by programmatically scanning each protein family tree, and identifying duplication and speciation events using a modified version of the species overlap algorithm [23]. As this is a critical step, commonly affected by phylogenetic inconsistencies and artifacts, we used an *ad hoc* algorithm to make speciation and duplication event detection tolerant to potential outliers and spurious sequences. This noise-tolerant algorithm was particularly necessary to reduce the number of false or dubious duplication events identified in bacterial and archaeal phylogenies, which typically produce numerous false OGs. The software implementing this method, as well as further details and benchmarking results based on the Quest for Orthologs standard framework [27], are available at (https://github.com/AnaHrnndz/pbdood).

### Hierarchically consistent and evolutionarily dated OGs

One of the main challenges of previous versions of eggNOG was achieving broad taxonomic coverage and predicting OGs at different taxonomic levels, while simultaneously ensuring the hierarchical consistency of the inferred OGs. Under the new pipeline, all evolutionarily related OGs are derived from the same phylogenetic tree, ensuring full hierarchical consistency. Furthermore, the putative evolutionary origin of each OG is taxonomically determined based on the LCA inferred for the duplication node in the tree from which it originated. This, together with ability to visually explore the OG phylogenetic placement and in-line functional annotations of the new eggNOG website interface, allows users to investigate the evolutionary path of particular proteins and functions.

### Improved OG size distribution in eggNOG v7

As eggNOG v7 uses the same core proteome dataset as eggNOG v6, we evaluated the performance of the new algorithm in producing more realistic OG sizes according to the number of species covered.

We applied the new pipeline to 59 310 557 sequences from 12 535 species, producing an initial set of 1 208 328 protein cluster families: 35 072 were Pfam domain-based, while 1 173 256 were generated by *de novo* clustering. We inferred phylogenies for each cluster and performed OG detection, identifying a total of 3 182 553 OGs at various taxonomic levels. Of these, 1 234 929 were identified as basal OGs, meaning that no other OG from the same phylogeny contains them.

**Figure 1.**
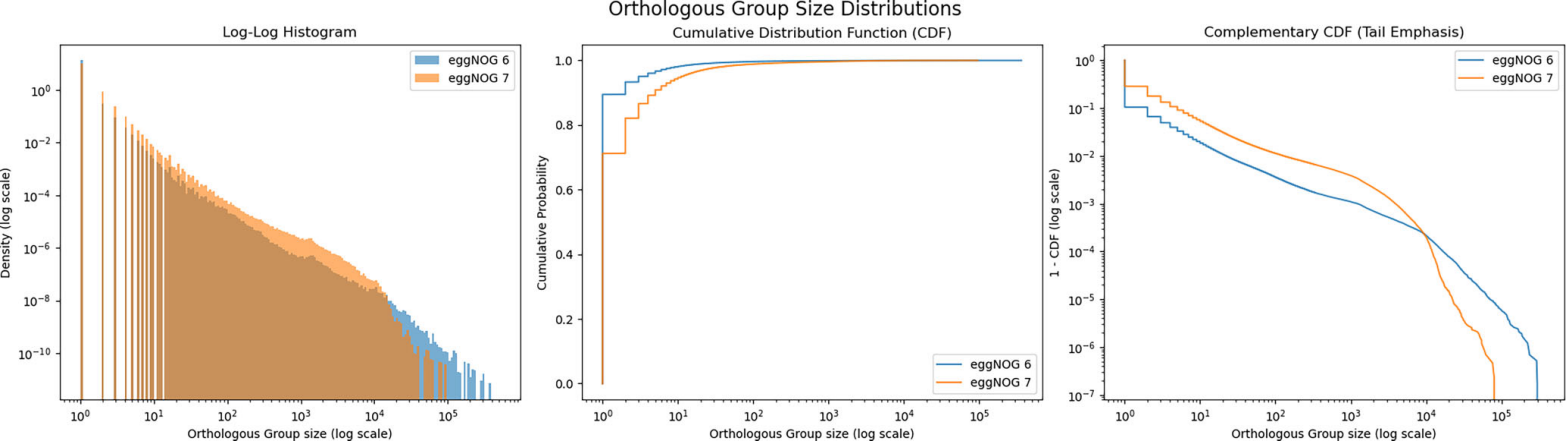
Size distributions of OGs in eggNOG versions 6 and 7. The left panel shows a log–log histogram of OG sizes, highlighting that eggNOG v7 reduces both the number of singletons (first bar on the left) and extremely large OGs (tail of the distribution) compared to eggNOG v6. The middle panel presents the cumulative distribution function (CDF) of OG sizes, showing the overall reduction in cluster fragmentation in eggNOG v7. The right panel shows the complementary CDF, focusing on the tail of the OG size distribution, where eggNOG v7 contains fewer large OGs.

We then compared the size distribution of the basal eggNOG v7 OGs to that of the eggNOG v6 LUCA taxonomic level, which was built using the same set of proteins. As shown in Fig. 1, the new OG delineation pipeline drastically reduced both the number of singletons (from 5 million orphan proteins to 3 million) and the number of OGs of extremely large size.

Taking into account the number of species covered (12 535), the distribution of OGs in eggNOG v7 is more biologically plausible overall. For example, when we calculated the number of duplication events occurring within each OG cluster, we found an average of 1.02 duplications per OG in eggNOG v7, compared to 4.00 in eggNOG v6. This duplication rate was calculated only for OGs at basal taxonomic levels, excluding species-specific duplication events, and it should be considered a proxy for the number of in-paralogs observed with each OG. The reduction in the number of in-paralogs per OG in eggNOG v7 can be attributed to the division of very large groups into more compact orthology-only groups.

As expected, Pfam-based OGs were of medium size, containing an average size of 1143 sequences per OG, and encompassing the majority of functionally annotated sequences (77%). In contrast, *de novo* clusters produced significantly smaller OGs (average size of 10 sequences), likely representing species-specific functional specializations and accessory gene families that are currently unknown.

### OGs in eggNOG v7 are more functionally informative

From a functional perspective, OGs in eggNOG v7 exhibited greater functional consistency than in previous versions. Using per-sequence KEGG [28] mappings, we evaluated the ability of eggNOG’s nonsupervised pipeline to reproduce the manually curated KEGG functional orthologous clusters (KOs).

**Figure 2.**
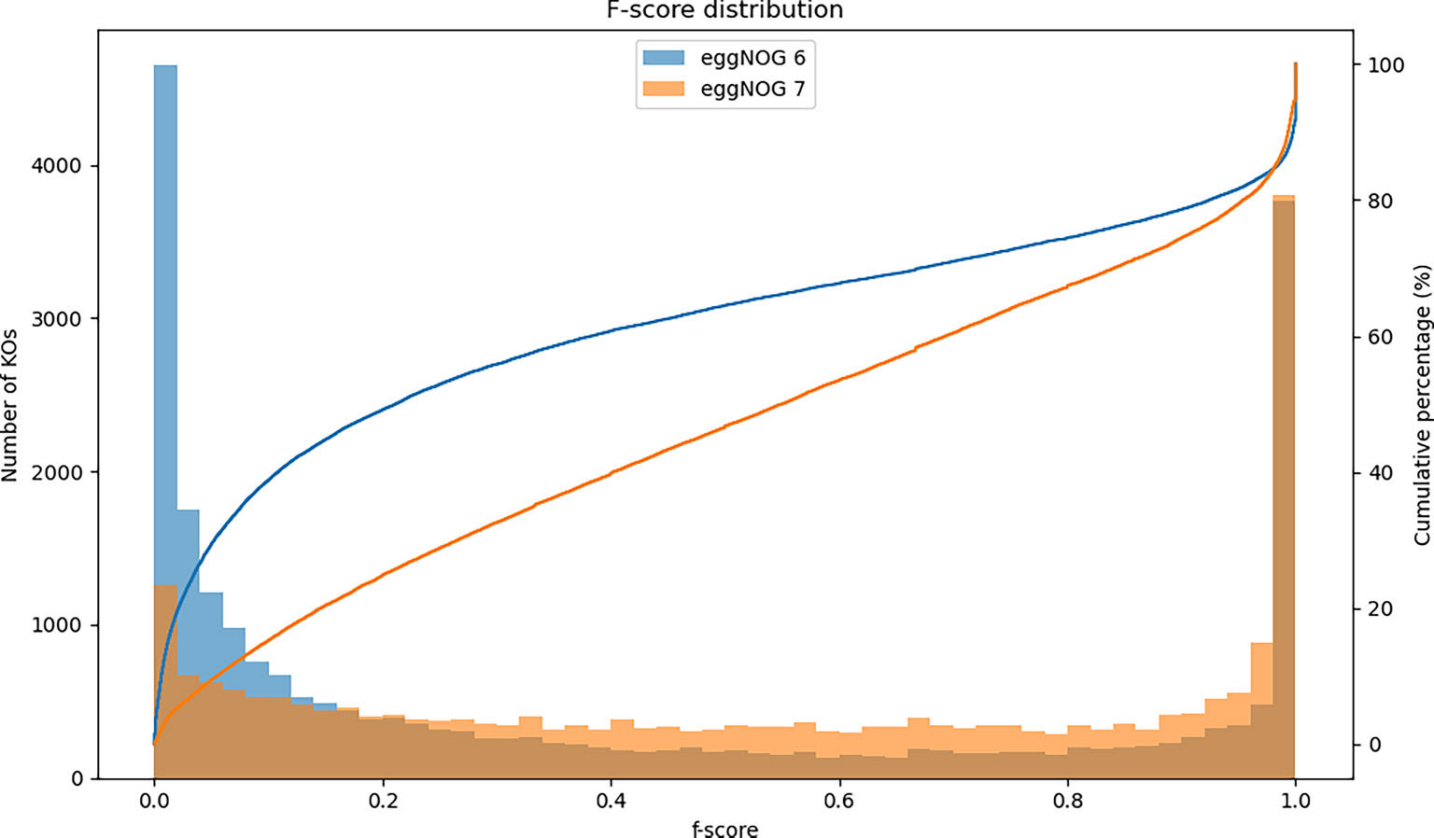
*F*-score distribution of functional OGs in eggNOG versions 6 and 7. Histograms show the distribution of *F*-scores for eggNOG OGs overlapping KEGG functional OGs (KOs) in versions 6 (blue) and 7 (orange). Solid lines indicate the cumulative distribution of *F*-scores for each version. Compared to eggNOG v6, eggNOG v7 exhibits a marked shift toward higher *F*-scores, reflecting greater functional consistency and improved agreement with manually curated KEGG KOs.

To perform the benchmark, we calculated the precision, recall, and *F*-score for each OG that shared at least one sequence with the KEGG database in both versions 6 and 7 of eggNOG. To avoid potential redundancy caused by the nested structure of OGs, where basal OGs can be subdivided into smaller groups at different taxonomic levels, we only included basal OGs in the comparisons.Overall, eggNOG v7 achieved a higher mean *F*-score (0.5393) than eggNOG v6 (0.3868), demonstrating improvement across all OG size categories (Fig. 2).

Furthermore, all protein annotations included in eggNOG v7 have been updated, providing up to date functional terms and links to external databases such as UniProt [29], Gene Ontology [30], COG [5], and KEGG [28]. We expect the new OGs and updated functional annotations to impact positively on functional profiling methods such as eggNOG-mapper [1].

## Website improvements

Previous versions of the eggNOG website focused on displaying the functional and evolutionary information of each OG. While informative, user feedback indicated that this interface was overly technical for most non-bioinformaticians. For example, most searches in our records involved protein names from model species rather than OG identifiers. However, although protein identifiers were always accepted as valid queries, the results page of previous eggNOG versions did not allow users to locate their query proteins within OGs, nor were able to show the hierarchical relationships among matching OGs. This made it difficult to use eggNOG to classify and annotate specific proteins, as well as to interpret their evolutionary path and putative functional specialization events.

**Figure 3.**
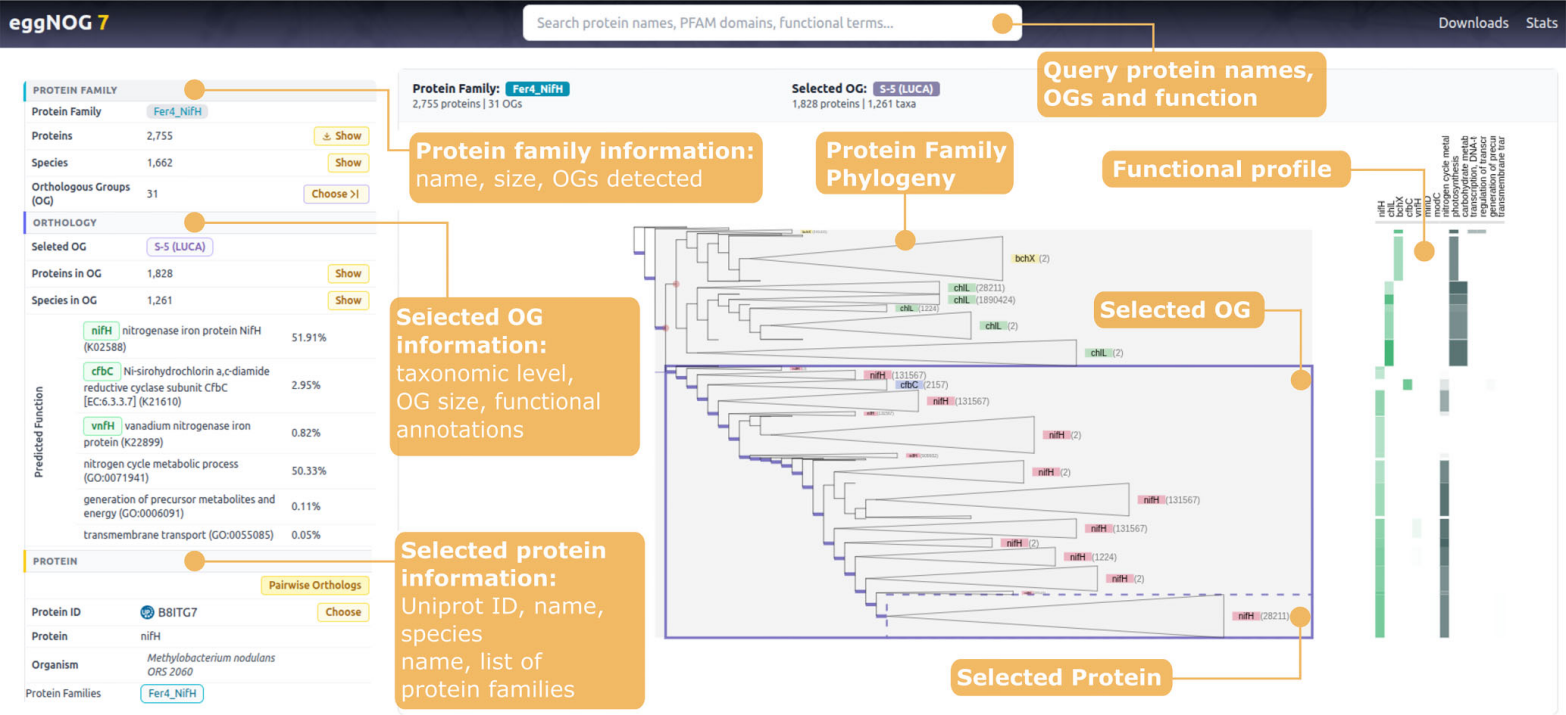
The redesigned eggNOG v7 web interface. The new interface provides an integrated, protein-centric view of orthology and function. Users can query protein names, OGs, and functions through the search bar. Results display detailed protein family information (name, size, and OGs detected), phylogenetic context, and functional profiles. The interface highlights selected OGs (with taxonomic level, OG size, and functional annotations) and selected proteins (with UniProt ID, name, species, and family membership). The phylogenetic tree view enables exploration of OG evolutionary history, while the functional profile panel integrates functional annotations for interactive navigation.

In eggNOG v7, we completely redesigned the web application to provide more intuitive workflows and interactions (Fig. 3). The new search panel provides instant access to protein, function, and OG names and identifiers, displayed separately for clarity. Users can search UniProt accessions, protein names, HGNC gene names, COG identifiers, and KEGG KO symbols, enabling easy cross-referencing with commonly used genomic and functional databases. The search now prioritizes genes and proteins from model species, particularly those included in the Alliance of Genome Resources [31].

When users query a specific protein, the left-hand panel shows all protein families it belongs to. For multidomain proteins, the system selects the largest OG detected by default, while the OG navigation panel lets users explore all other related OGs and families. In addition, a “Pairwise orthology” button generates an aggregated list of orthologous proteins in other organisms from all relevant OGs and taxonomic levels, which users can easily explore and download.

The new right-hand panel displays the phylogeny used to identify the selected OG, highlighting the placement of the queried protein. This interface uses the latest ETE toolkit [25] to dynamically explore very large trees (over 100k sequences) with a custom eggNOG layout. Most common functional terms for each tree branch, as well as their taxonomic scope are also dynamically shown in the tree. Furthermore, eggNOG now uses TreeProfiler [32] to build functional profiles of KEGG KO symbols and Gene Ontology terms [30] across all the tree branches. This generates a dynamic heatmap of presence–absence terms that allows users to link evolutionary events (e.g. gene duplications) to subfunctionalization events. Protein domain architecture and other sequence-based features can be visualized along the tree by activating additional layouts.

Conversely, users can perform a reverse search by querying terms that refer to general functions or protein domains (e.g. generic gene names, KEGG KO symbols, or Gene Ontology terms). In this search mode, the left-side panel dynamically updates to list all protein families, OGs, and protein sequences that match the queried term across multiple species. This functionality is particularly useful for investigating the taxonomic distribution and evolutionary history of specific functions or domains.

## Data Availability

All data are available through web queries and as bulk downloads at https://eggnogdb.org. Downloadable files include multiple sequence alignments and phylogenetic trees for all protein families, OG information, and functional annotation datasets.
